# Whole genome sequences of eight *Erwinia amylovora* phages isolated from South Korea

**DOI:** 10.1128/mra.01062-24

**Published:** 2025-02-25

**Authors:** Eunjung Roh, Madison E. Duffy, Leslie M. Ewool, Julianne H. Grose

**Affiliations:** 1 Crop Protection Division, National Institute of Agricultural Sciences, Rural Development Administration, Wanju-gun, South Korea; 2 Department of Microbiology and Molecular Biology, Brigham Young University, Provo, Utah, USA; DOE Joint Genome Institute, Berkeley, California, USA

**Keywords:** bacteriophages, *Erwinia amylovora*, Fifi318, Fifi44, Fifi011, Fifi051, Fifi067, Fifi106, Fifi287, Fifi450

## Abstract

The gram-negative *Enterobacteriaceae Erwinia amylovora* is the causative agent of fire blight. Herein, we announce the full genome sequencing and annotation of eight *E. amylovora* bacteriophages from apple and pear orchards in South Korea, which have remarkable similarity to *Erwinia* phages previously isolated from the Americas.

## ANNOUNCEMENT

*Erwinia amylovora,* a gram-negative *Enterobacteriaceae*, is the devastating causative agent of fire blight (1), the most destructive disease affecting apple and pear trees worldwide. The widespread use of antibiotics, such as streptomycin, in the treatment of fire blight has resulted in the emergence and spread of resistant strains (2, 3). Bacteriophages have been shown to be effective alternative treatments for fire blight (4–6); however, there have been few studies on strain variation (7–9), as well as phage specificity for these strains (10), which may render therapies ineffective.

Herein, we announce the whole genome sequence and annotation of eight phages that infect *E. amylovora* YBK14808 (NZ_CP14766

0), isolated from soil or water collected from apple and pear orchards in South Korea in September 2020 (*Erwinia* phages Fifi011, Fifi44, Fifi051, Fifi067, Fifi106, Fifi287, Fifi318, and Fifi450). Phages were isolated using tryptic soy broth/agar by incubating an overnight culture of YBK14808 with topsoil or water for 24–48 hours. These enrichment cultures were centrifuged, and the supernatant was incubated with an overnight culture of YBK14808 and then plated in top agar. The resulting plaques were submitted to three rounds of plaque purification as described (11).

Bacteriophage DNA was isolated from the lysate using the Norgen Biotek Phage DNA Isolation Kit (Canada) and prepared for 151 bp paired-end Illumina MiSeq sequencing (except for phage Fifi011, which was run on iSeq) using the NEBNext Ultra II DNA Library Kit (New England Biolabs, Ipswich, MA, USA). The resulting contigs were assembled using Geneious 2022 *de novo* assembly (12) and were annotated using DNA Master 5.23.6 (13), GeneMarkS 4.25 (14), and BlastP (15) of the non-redundant protein sequences database. Default parameters were used for all programs, including quality control trimming and assembly in Geneious.

All eight phage genomes circularized upon assembly, consistent with complete genomes, and were assigned to three previously described *Enterobacteriaceae* phage clusters by whole genome Gepard dot plot (16) alignment over 50% of the genome as described (17). Phages Fifi011, Fifi051, Fifi067, Fifi287, and Fifi450 have genomes ranging from 74,152 to 76,093 bp and show dot plot homology to N4-like phages. BlastN (15) taxonomy indicates that they belong to the *Caudoviricetes* family *Schitoviridae*, with Fifi011 being an unknown *Waedenswilvirus* and the other four phages *Yonginviruses*. Phages Fifi106 and Fifi318 (84,284 and 84,539 bp, respectively) exhibit dot plot homology to the *Enterobacteriaceae* FelixO1-like cluster and belong to the *Kolesnikviruses* of the *Ounavirinae* family, as determined by BlastN. Fifi44 (53,436 bp) shows dot plot homology to the *Enterobacteriaceae* øEcoM-GJ1-like phages and belongs to the *Chaseviridae* family, *Cleopatravirinae* subfamily according to BlastN. PhageTerm (18) predicted headful packaging for Fifi44, consistent with its T1-like TerL (19), thus its operon with TerL was assigned bp1. The TerL encoded by the N4-like phages is homologous to N4’s TerL, which packages DNA with short direct terminal repeats (sDTRs) (20), and PhageTerm (18) confirmed sDTRs from raw sequencing reads from both Fifi067 and Fifi287. Analysis of the FelixO1-like phages Fifi106 and Fifi318 by PhageTerm (18) also suggested sDTRs. All seven putative sDTR phages, based on TerL homology (21) and Phageterm of the relatives described above, were assigned bp1 from the rearranged genome downloaded from Phageterm.

**TABLE 1:** Genomic properties of eight *Erwinia amylovora* phages isolated from apple and pear orchards in South Korea^*b*^

Phage name	GenBank accession no.	SRA accession no.	Total no. of reads	Fold coverage range (mean)	bp	GC%	Isolation source (type, orchard)	GPS	Closest relative (Tax.)^*a*^
Fifi011	PQ051109	SRR30767472	122,380	221.54	74,152	52.60	Soil, pear	37° 00′38.99″ N 127° 16′13.01″ E	EamP-S6 (22) (W)
Fifi44	PQ051110	SRR30767471	177,182	467.07	53,436	43.95	Soil, pear	37° 00′38.99″ N 127° 16′13.01″ E	SNUABM_27 (23) (L)
Fifi051	PQ072212	SRR30767470	37,735	69.52	76,093	46.91	Soil, pear	37° 00′38.99″ N 127° 16′13.01″ E	EaP-8 (24) (Y)
Fifi067	OP829055	SRR30767469	66,292	122.63	75,184	48.07	Water, apple	36° 58′ 52.6944″ N 127° 56′ 9.2580″ E	Kuerle (Y)
Fifi106	OR284297	SRR30767468	59,472	97.99	84,282	43.39	Soil, apple	36° 58′ 52.6944″ N 127° 56′ 9.2580″ E	Hena2 (25) (K)
Fifi287	PQ051113	SRR30767467	100,262	183.66	74,892	48.10	Water, pear	37° 00′38.99″ N 127° 16′13.01″ E	Kuerle (Y)
Fifi318	PQ051114	SRR30767466	52,119	85.46	84,539	43.83	Water, apple	36° 58′ 52.6944″ N 127° 56′ 9.2580″ E	Pistou (K)
Fifi450	PQ051115	SRR30767465	44,560	82.96	74,913	48.02	Water, pear	37° 00′38.99″ N 127° 16′13.01″ E	Kuerle (Y)

^*a*^ Closest relative and taxonomies (Tax.) were from BLASTN output. Phage references are provided when available (Kuerle OQ18121 isolated by Yu et al. and Pistou OQ818706 isolated by Garneau et al. were unpublished). For taxonomy, both “Y” and “W” belong to the *Caudoviricetes; Schitoviridae*; with “Y” standing for *Erskinevirinae; Yonginvirus*; unknown *Yonginvirus* and “W” standing for *Waedenswilvirus*; unknown *Waedenswilvirus*. “K” is for *Caudoviricetes; Ounavirinae; Kolesnikvirus*; unknown *Kolesnikviru*s, and “L” stands for *Caudoviricetes; Chaseviridae; Cleopatravirinae; Loessnervirus*; unknown *Loessnerviru*s.

^*b*^ Phage name, GenBank and SRA accession number, total number of sequencing reads used for assembly, fold coverage, genome length, GC percentage, isolation source (water or soil and orchard type), GPS, and suggested taxonomy are provided.

## Data Availability

GenBank whole genome accession numbers and raw sequence data (SRA accession numbers) are provided in Table 1.
